# Tracheostomy and mechanical ventilation weaning in children affected by respiratory virus according to a weaning protocol in a pediatric intensive care unit in Argentina: an observational restrospective trial

**DOI:** 10.1186/1824-7288-37-5

**Published:** 2011-01-19

**Authors:** Gustavo Caprotta, Patricia Gonzalez Crotti, Judith Frydman

**Affiliations:** 1 MD, Pediatric Critical Care physician, Head of Pediatric Intensive Care Unit of "Hospital de Trauma y Emergencia Dr. Federico Abete", Malvinas Argentinas Buenos aires, Argentina; 2 MD, Pediatric Critical Care physician. Staff and Emergency doctor at "Hospital de Trauma y Emergencia Dr. Federico Abete de Malvinas Argentinas", Buenos Aires, Argentina; 3 Bachellor in Respiratory Therapy in intensive care. Coordinator of Respiratory Therapy in the Pediatric Intensive Care Unit at "Hospital de Trauma y Emergencia Dr. Federico Abete de Malvinas Argentinas", Buenos Aires, Argentina

## Abstract

We describe difficult weaning after prolonged mechanical ventilation in three tracheostomized children affected by respiratory virus infection. Although the spontaneous breathing trials were successful, the patients failed all extubations. Therefore a tracheostomy was performed and the weaning plan was begun. The strategy for weaning was the decrease of ventilation support combining pressure control ventilation (PCV) with increasing periods of continuous positive airway pressure + pressure support ventilation (CPAP + PSV) and then CPAP + PSV with increasing intervals of T-piece. They presented acute respiratory distress syndrome on admission with high requirements of mechanical ventilation (MV).

Intervening factors in the capabilities and loads of the respiratory system were considered and optimized. The average MV time was 69 days and weaning time 31 days.

We report satisfactory results within the context of a directed weaning protocol.

## Introduction

During winter in 2009, Argentina as well as several other countries in the world, was affected by Influenza A H1N1 pandemic [1].

Our Pediatric Intensive Care Unit (PICU) was the referral center for this pandemic in the province of Buenos Aires.

Between June 1 and July 31 2009 113 patients were admitted in our PICU, 79 of them with respiratory pathology. Twenty presented positive PCR (polymerase chain reaction) by Influenza A H1N1and 22 presented positive IFI (indirect immunofluorescence) by RSV (respiratory syncitial virus). Out of the 69 patients who survived 3 presented difficult weaning and needed tracheostomy.

This paper describes the clinical behavior and presentation of these 3 patients who were admitted into our PICU in that period and who had a prolonged stay due to respiratory complications after viral infection (Table 1).

**Table 1:** Patient characteristics

	Case 1	Case 2	Case 3
Age	6 months	6 months	1 month 20 days

Gender	Male	Male	Female

Socioeconomic level	Overcrowding	Overcrowding	Low

Mother's level of education	Incomplete Primary School	Complete High School	Incomplete High School

Immunization schedule	Complete	Incomplete	Complete

Previous disease history	Genetic sme. Prematurity Malnutrition	Healthy	Healthy

Days of previous symptoms	5 days Fever and cough	5 days Rhinorrhea and cough	2 days Cough and rhinorrhea

Diagnosis on admittance	Bilateral Pneumonia	Bronchiolitis	Pneumonia RUL

Pafi on admittance	118	49	92

Mean Map	15.2 (5.8-23.8)	17.2 (8.8-24.4)	9.8 (2-16.2)

Chest X-ray on admittance	Bilateral diffuse infíltrate	Bilateral diffuse infiltrate	Focal condensation RUL

VTSNF	H1N1	H1N1 RSV	H1N1 RSV

MV from admittance date	Yes	Yes	Yes

ARDS from admittance date	Yes	Yes	Yes

Overcrowding: defined as the condition caused by the cohabitation of three or more people per room.

Sme: Syndrome

RUL: Right Upper Lobe

RSV: Respiratory Syncytial Virus

H1N1: Influenza A (H1N1) Virus

VTSNF: Virological Test for Nasopharyngeal Secretion

MV: Mechanical Ventilation

ARDS: Acute Respiratory Distress Syndrome

Pafi: O2 arterial pressure/O2 inspired fraction

Map: Mean airway pressure

We report the results obtained according to a directed weaning protocol.

Below, we describe each patient's evolution before tracheostomy was performed and before the weaning plan was begun.

### Case 1

A six-month-old male patient was admitted in our PICU after intubation owing to respiratory insufficiency due to bilateral pneumonia. Through nasopharyngeal secretions a positive PCR by Influenza A H1N1 virus was found. He was a 28-week preterm infant with a birth weight of 900 grams. He had bronchopulmonary dysplasia (BPD) because of mechanical ventilation during 2 months in the perinatal age. He also presented some malformations compatible with a genetic syndrome which was still being studied before admission (retromicrognathia, abdominal angioma in the medial line, low implantation of the ears, among others).

**Figure 1:**
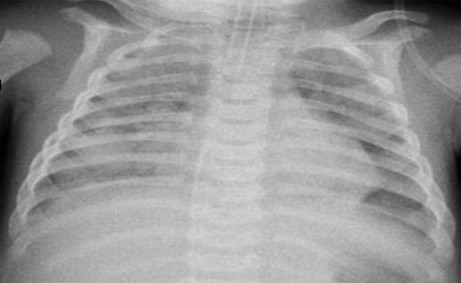
**Chest X-ray of case 1**.

On admission the patient presented severe acute respiratory distress syndrome (ARDS) for which he required high MV parameters (Figure 1). He presented serious refractory hypoxemia and remained 36 days on MV. Using a low tidal volume ventilation protocol we were not able to achieve an adequate minimal oxygenation. So, as we do not count on high frequency oscillatory ventilation (HFOV) because of certain socio-economic limitations within our hospital, we resorted to standard modalities, reaching mean airway pressure (Paw) of 15.2

Extubation failed by respiratory insufficiency in 2 opportunities requiring reintubation in the first 24 hours. In both opportunities the patient had passed a spontaneous breathing trial (SBT).

A respiratory endoscopy was performed which showed an inflammatory pathology of the upper airway (UA).

These findings together with presenting prolonged MV (36 days) led to the decision of carrying out a tracheostomy and of beginning a weaning plan.

### Case 2

A six-month old male patient born in due term with adequate weight, previously healthy. He was referred to our PICU for presenting bronchiolitis by co-infection with RSV and A H1N1 virus.

**Figure 2:**
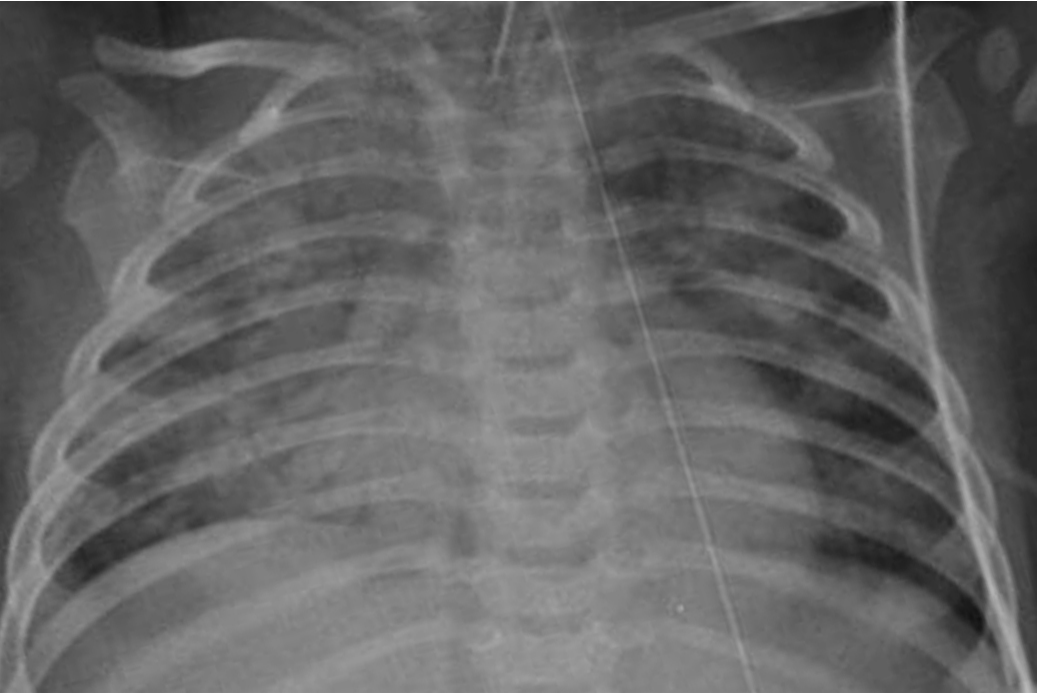
**Chest X-ray of case 2**.

He presented severe ARDS and hypoxemia and required high MV parameters remaining ventilated for 51 days (Figure 2). Three SBTs were performed during his evolution with 3 elective extubations, failing in all cases. He also presented failure during disconnection from positive pressure with non-invasive positive pressure ventilation (NPPV) due to respiratory insufficiency.

A respiratory endoscopy was performed which showed an important edema of the larynx crown and posterior synechia due to which tracheostomy was performed on the 44^th ^day of hospitalization. Then, the weaning plan was started.

### Case 3

**Figure 3:**
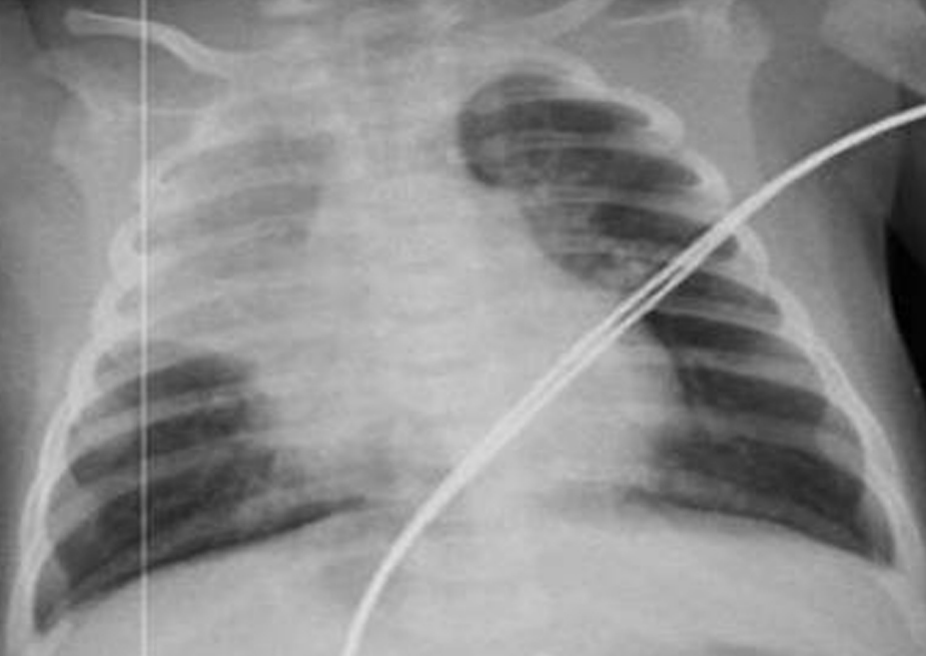
**Chest X-ray of case 3**.

A female patient of 1 month and 20 days of age, born in due term with adequate weight, with no perinatologic history. She was admitted with low acute respiratory failure following pneumonia in the right superior lobe. Co-infection with RSV and Influenza A H1N1 virus was found. During her prolonged stay in PICU (111 days) she required MV due to ARDS with severe hypoxemia (Figure 3).

In five opportunities she passed an SBT but in all cases re-intubation was required within 48 hs owing to low respiratory involvement. Also, repeated presence of condensation in the right superior lobe was observed. Due to everything mentioned above, a respiratory endoscopy was performed, reporting a right anomalous tracheal bronchus and mild subglottic edema. Given the fact that the patient had several respiratory infections which led to an obstruction of the segment corresponding to the anomalous bronchus, an exeresis of the right anomalous tracheal bronchus and a segmentectomy of the right superior lobe were performed.

After 51 days on MV a tracheostomy was performed and respiratory weaning was started. Disconnection of positive pressure was achieved after 98 days.

The 3 patients described coincided in positive PCR for Influenza A virus (2 of them with co-infection with RSV), prolonged MV, ARDS with severe hypoxemia and successful SBTs with failed extubations during the course of 48 hours.

Those patients presented upper airway pathology by pathologic endoscopy and lower airway pathology (post viral sequelae). These two factors affected their weaning from MV.

In our PICU upon meeting the following criteria we initiate SBT based on current consensus: resolution of the basal cause of respiratory failure, peak inspiratory pressure (PIP) < 25 cm H_2_O, positive end-expiratory pressure (PEEP) < 5 cm H_2_O, tidal volume 6-8 ml/kg, FIo_2 _< 0.40, intermittent and decreasing sedation, patient alert, hemoglobin > 10 g/dl, and decreasing vasoactives [2-4].

In our PICU SBTs are performed with a T-piece and supervised by respiratory therapists. The variables to be considered are registered on charts that we have developed for that purpose and which are filled in at the start of the test and every 15 minutes during the two hours the test lasts.

It is PICU's policy to perform respiratory endoscopies in those children who present two or more failed extubations.

In all three cases, because of presenting a pathologic endoscopy the decision of performing a tracheostomy was discussed and accepted by the patients' parents. All of them were trained in the handling, hygiene and comfort of the tracheostomy.

For the patients we presented, a strategy was designed with the objective of achieving disconnection from positive pressure by means of an orderly and progressive protocol.

In the first place we measured the maximum tolerated time on CPAP in each patient. We have defined maximum time as the longest period the patient is able to remain on CPAP without altering his vital signs in +/- 20%. This determination was carried out comparing the physiologic values found at the beginning of the evaluation and performing their follow-up every 15 minutes.

The mean value of the maximum tolerated time on CPAP was 4 hours (3-6 hours).

Out of the maximum time reached, between 50 and 75% was used for the respiratory training of the patient.

The objective was to train the patient without exposing him to fatigue and muscular tiredness due to the decrease in his respiratory capacitance. This allowed to program rest periods which became less frequent as training progressed. This plan was carried out during day time while during the night, sleeping time was respected supplying MV in PCV [5].

**Figure 4:**
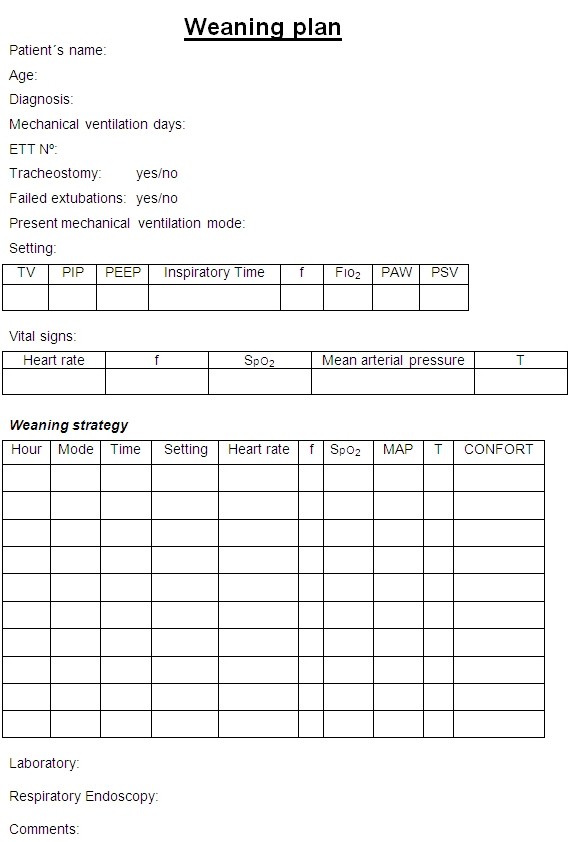
**Chart of weaning**. EET: Endotracheal tube TV: Tidal Volume PIP: Peak inspiratory pressure PEEP: Positive end espiratory pressure f: Respiratory rate FIO2: fraction of inspired oxygen PAW: medium airway pressure PSV: Pressure support ventilation SpO2: oxygen partial saturation T: temperature MAP: mean arterial pressure

A gradual diminution of the ventilatory support, combining PCV with increasing periods on CPAP + PSV, was performed. Once the patient managed to remain 12 hours on CPAP + PSV, this modality was alternated with T-piece whose duration was progressively incremented [6] (Figure 4).

The objectives of the programed tasks for the day were described in a chart in which the physiological parameters found during the training period were constantly recorded: respiratory rate, heart rate, blood pressure, oxygen saturation, maximal inspiratory pressure (PIMAX), maximal expiratory pressure (PEMAX), ventilatory mechanics, use of accessory muscles, comfort index and rapid shallow breathing index (respiratory rate/tidal volume per kg of weight)[2].

For the measurement of PIMAX and PEMAX a Marshalltown ™ manual vacuum meter was connected to the endotracheal tube. We selected the most negative and positive value of 3 efforts during spontaneous breathing over a period of 20 seconds occluding the airway with a one-way valve [2,3].

In case the patient presented signs of muscular fatigue which kept us from accomplishing the programmed task we returned to the work plan of the previous day (when he tolerated the exercise adequately) and he was re-assessed the following morning with the same parameters.

With this muscular training, nutritional support and the supply of vitamins and minerals, an improvement of the respiratory mechanics was observed, focusing on the measured parameters managing to effectively disconnect patients from positive pressure [7]. This objective was considered as accomplished when the patient remained on a T-piece over a period of 48 hs.

The average MV time was 69 days. The average weaning time was 31.

Finally, the 3 patients were referred to a general pediatric ward, two of them with supplementary oxygen.

## Discussion

Children with severe respiratory disease due respiratory virus presented a high percentage of ARDS on admittance to PICU with severe hypoxemia, requiring high MV parameters. Among the surviving patients, a torpid evolution with long periods on MV and difficulties for its disconnection were observed [8].

Unfortunately, up to date there aren't available protocols based on evidence to guide a difficult weaning process in tracheostomized children. The bibliography on adults is ample, but not always applicable to our infant population due to anatomic, functional and cognitive-affective reasons [9].

Due to the functional anatomic differences between children's and adults' air way, the ideal time to indicate tracheostomy after a prolonged period on MV is still being discussed [10]. This is why, in the bibliography, more prolonged periods on MV through an endotracheal tube (ETT) are found in children than in adults.

Tracheostomy facilitates weaning with respect to ETT because it produces: dead space reduction, less airway resistance, decreased breathing work, better secretion removal with suctioning, less likelihood of tube obstruction, improved patient comfort, less need for sedation and better glottic function with less risk of aspiration [5,6,11].

The term weaning refers to the transition from ventilatory support to completely spontaneous breathing, during which time the patient assumes the responsibility for effective gas exchange while positive pressure support is withdrawn [12].

Weaning consumes between 40 and 60% of mechanical ventilation time. About 25% of ventilated patients present difficulties in the disconnection from the ventilator, being unable to be disconnected by means of SBT, requiring a progressive procedure which can take several days, weeks or even months [13]. These patients would benefit from a directed weaning protocol.

Ventilatory failure may be generated by a deterioration of the neuromuscular capacity or by increasing the loads of the respiratory system. The decrease of the capacity may be owed to a depression of the respiratory center (whether by sedation, alkalosis or encephalic lesion), neuropathies (polyneuropathy of the critical patient), muscular disorders (malnutrition, hyperinsuflation, electrolyte disorders and the use of muscle relaxants and corticoids) and chest wall abnormality (unstable chest, post-operative pain). The increase in the load may be due to the high ventilation requirements (sepsis, anxiety and pain), resistive loads (bronchospasm, secretions), elastic loads (auto PEEP, decrease in compliance) and related to the ventilator and the endotracheal tube (valves, small tubes) [6,12].

All these factors were considered and optimized in our patients before proceeding to their extubation.

Among the elements we count on, we opted for the use of the described combination of ventilatory modalities as part of an orderly and progressive protocol. This permitted the favoring of the muscular training, the improvement of the patient-ventilator interaction and therefore the enhancement of the respiratory work.

The use of PCV enabled us to ensure total ventilation during night rest, eliminating energetic waste used in training which allowed the recovery.

PSV allowed the patient to control his own frequency and therefore the circulating volume and minute volume. In this manner the PSV generated an adequate respiratory work and oxygen consumption of the respiratory muscles, diminishing fatigue and favoring re-training [12].

The combination of this modality with CPAP allowed to increase the functional residual capacity thus achieving a better oxygenation. This is owing to the increase in the number of effective alveolar units in gas exchange (alveolar recruitment). CPAP is able to offset the abnormal closing of the airway and the effect of the auto PEEP, noticing the beneficial effects when the level of inspiratory efforts to be developed during spontaneous breathing diminishes.

The use of a T-piece allowed for greater independence from the ventilator with less resistance in the airway since the opening of an inspiratory valve is not necessary. In addition it permitted a clinical evaluation of the diaphragmatic function which was not possible if the patient received any kind of inspiratory support [14].

## Conclusion

The three described patients had pulmonary sequelae for infection caused by respiratory virus with a pathology of the airway evidenced by respiratory endoscopy, and in spite of all this, their effective disconnection from positive pressure was achieved. This was possible combining tracheostomy with an orderly, monitored and progressive muscular training plan (directed weaning protocol).

Even though more studies are necessary to develop weaning protocols based on evidence, we believe that this type of approach may be useful in pediatric patients with difficult weaning due to other etiologies.

## Competing interests

The authors declare that they have no competing interests.

## Authors' contributions

GC participated in the writing of the manuscript, design of the study and performed the statistical analysis. PGC participated in the writing of the manuscript and data collection. JF participated in the writing of the manuscript, conceived of the study and data collection. All authors read and approved the final manuscript.

## Consent

Written informed consent was obtained from the parents of the patient for publication of this report and accompanying images. A copy of the written consent is available for review by the Editor-in-Chief of this journal.
